# Dyspepsia

**Published:** 1855-11

**Authors:** 


### DYSPEPSIA.

THE nervous energy is the motive power of the whole man, spiritual, mental and physical. When that power is equally distributed, the body is well, the brain is clear, and the heart is buoyant. If the brain has more than its share, it burns itself up, and makes the "lean *Cassius*,"—the restless body and the anxious countenance.

As there is a given quantity of nervous influence for the whole body, if the brain has more than its natural portion, the stomach has less, consequently the food is not thoroughly assimilated, or, as we call it, "*digested.*" This being the case, the requisite amount of nutriment is not derived from the food, and the whole body suffers, doubly suffers; for not only is the supply of nutriment deficient, but the quality is imperfect. These things go on,—aggravating each other, until there is not a sound spot in the whole body; the whole machinery of the man is by turns the seat of some ache or pain, or "symptom." This is a common form of aggravated dyspepsia.

Such being the facts, some useful practical lessons may be learned.

1. Never sit down to table with an anxious or disturbed mind; better a hundred-fold intermit that meal, for there will then be that much more food in the world for hungrier stomachs than yours; and besides, eating under such circumstances can only, and will always, prolong and aggravate the condition of things.

2. Never sit down to a meal after any intense mental effort, for physical and mental injury is inevitable, and no man has a right deliberately to injure body, mind or estate.

3. Never go to a full table during bodily exhaustion; designated by some as *being worn out*, *tired to death*, *used up*, *done over*, and the like. The wisest thing you can do under such circumstances, is to take a cracker and a cup of warm tea, either black or green, and no more. In ten minutes you will feel a degree of refreshment and liveliness, which will be pleasantly surprising to you; not of the transient kind, which a glass of liquor affords, but permanent, for the tea gives present stimulus and a little strength, and before it subsides, nutriment begins to be drawn from the sugar and cream and bread, thus allowing the body, gradually and by safe degrees, to regain its usual vigor. Then, in a couple of hours you may take a full meal, provided it does not bring it later than two hours before sundown; if later, then take nothing for that day, in addition to the cracker and tea, and the next day, you will feel a freshness and vigor not recently known. No reader will require to be advised a second time, who will make a trial as above, while it is a fact of no unusual observation among intelligent physicians, that eating heartily, under bodily exhaustion, is not an infrequent cause of alarming and painful illness, and sometimes of sudden death. These things being so, let every family make it a point to assemble around the family board with kindly feelings, with a cheerful humor and a courteous spirit; and let that member of it be sent from the table in disgrace, who presumes to mar, the ought to be, blest reunion, by sullen silence, or impatient look, or angry tone, or complaining tongue. Eat in thankful gladness, or away with you to the kitchen, you graceless churl, you ungrateful, pestilent lout that you are. There was grand and good philosophy in the old time custom of having a buffoon, or music, at the dinner-table.

